# Effects of *Lacticaseibacillus paracasei* Strain Shirota on Daytime Performance in Healthy Office Workers: A Double-Blind, Randomized, Crossover, Placebo-Controlled Trial

**DOI:** 10.3390/nu15245119

**Published:** 2023-12-15

**Authors:** Hiroko Kikuchi-Hayakawa, Hiroshi Ishikawa, Kazunori Suda, Yusuke Gondo, Genki Hirasawa, Hayato Nakamura, Mai Takada, Mitsuhisa Kawai, Kazunori Matsuda

**Affiliations:** 1 Yakult Central Institute, 5-11 Izumi, Kunitachi-shi, Tokyo 186-8650, Japan; 2 Yakult Honsha European Research Center for Microbiology VOF, Technologiepark 94 bus 3, 9052 Ghent, Belgium

**Keywords:** probiotics, daytime performance, electroencephalograms, heart rate

## Abstract

*Lacticaseibacillus paracasei* strain Shirota (LcS) modulates psychological homeostasis via the gut–brain axis. To explore the possible efficacy of LcS for improving daytime performance, we conducted a double-blind, randomized, crossover, placebo-controlled study of 12 healthy office workers with sleep complaints. The participants received fermented milk containing viable LcS (daily intake of 1 × 10^11^ colony-forming units) and non-fermented placebo milk, each for a 4-week period. In the last week of each period, the participants underwent assessments of their subjective mood and measurements of physiological state indicators via an electroencephalogram (EEG) and heart rate variability in the morning and afternoon. The attention score in the afternoon as assessed by the visual analog scale was higher in the LcS intake period than in the placebo intake period (*p* = 0.041). Theta power on EEG measured at rest or during an auditory oddball task in the afternoon was significantly lower in the LcS period than in the placebo period (*p* = 0.025 and 0.009, respectively). The change rate of theta power was associated with the change in attention score. Treatment-associated changes were also observed in heart rate and the sympathetic nerve activity index. These results indicate that LcS has possible efficacy for improving daytime performance, supported by observations of the related physiological state indicators.

## 1. Introduction

Exposure to psychological stress causes various physical and mental disorders via the nervous and endocrine systems, such as depression, stomach pain, headaches, loss of appetite, diarrhea, fatigue, and insomnia [1,2,3]. Occupational stress and sleep disorders are associated with presenteeism, which refers to productivity losses from employees working with poor health conditions [4,5]. In Japan, costs corresponding to lost productivity due to presenteeism account for 80% of companies’ total health-related costs and are higher than the costs of absenteeism [6].

One of the symptoms of mental stress is disordered sleep, which has been found to increase cognitive task errors [7] and the risk of accidents [8]. This is because sleep disturbances can cause fatigue, drowsiness, decreased alertness, and lack of motivation, affecting daytime performance [7]. These mood indicators are commonly assessed by self-reported questionnaires. However, physiological state indicators such as electroencephalography (EEG) and heart rate variability (HRV) have been used as surrogate measures of daytime performance. EEG is a non-invasive technique for recording the brain’s electrical activity and reflects changes in mental status and cognitive function [9,10]. The EEG frequency bands beta (13–30 Hz), alpha (8–13 Hz), theta (4–8 Hz), and delta (0–4 Hz) are commonly used for studying brain activities. The P300 wave is an event-related potential (ERP) component elicited by the target stimulus in an oddball task occurring at around the 300 ms latency region and is commonly used to estimate cognitive performance. HRV analysis provides information about the status of the autonomic nervous system [11]. High-frequency (HF) components at 0.15–0.4 Hz reflect parasympathetic nerve activity, whereas the ratio of HF to low-frequency (LF) (0.04–0.15 Hz) components reflects sympathetic nerve activity [12].

The digestive system is highly regulated by the brain and state of mind, such as anxiety, depression, and fear, which greatly affect its functions. The brain and gut exchange information in both directions through the nervous, endocrine, and immune systems and are closely influenced by each other, forming a relationship called the gut–brain axis [13]. In recent years, the concept of a microbiota–gut–brain axis has been gaining attention as studies of stress [14,15,16] and mental illness [17,18,19] have produced a growing body of evidence that the gut microbiota is involved in the gut–brain axis. In addition, probiotics that contribute to the health of the host, either directly or through the improvement of the intestinal environment, are expected to exert positive effects via the gut–brain axis [20,21,22,23].

*Lacticaseibacillus paracasei* strain Shirota (LcS; formerly named the *Lactobacillus casei* strain Shirota [24]) is a widely used probiotic strain, which was selected from the microbial collection of Dr. Minoru Shirota, the founder of Yakult Honsha Co., Ltd. (Tokyo, Japan) LcS has a long history of safe use as a food material for over 80 years, and the United States Food and Drug Administration has accredited it as Generally Recognized As Safe (GRAS) [25]. LcS has been revealed in numerous clinical studies to have functions such as bowel movement normalization [26,27], infection prevention [28], and immunomodulation [29]. It has also been found to act on the nervous and endocrine systems. We previously reported that an LcS intervention in medical students preparing for an academic exam prevented the onset of physical symptoms and attenuated a stress-induced rise in salivary cortisol [30,31,32]. In addition, it was effective in maintaining the quality of sleep under academic stress conditions by suppressing the decline in deep sleep and shortening sleep latency, as measured by both subjective questionnaires and sleep EEG measures [33].

To explore the possible efficacy of LcS for improving daytime performance, we conducted a double-blind, randomized, crossover, placebo-controlled study in healthy participants with sleep complaints. We assessed their subjective mood using perceived mood questionnaires and measured physiological indicators via EEG and HRV.

## 2. Materials and Methods

### 2.1. Test Beverages

Fermented milk containing at least 1.0 × 10^11^ colony-forming units of LcS (strain no.: YIT 9029) per bottle (100 mL) was used as the test beverage. The nutritional content per bottle was as follows: 1.5 g of protein, 0.1 g of fat, 14.1 g of carbohydrates, and 63.0 kcal calories. The probiotic strain was obtained from the Culture Collection Research Laboratory of Yakult Central Institute, Tokyo, Japan. Since there is insufficient evidence for the efficacy of any probiotic strain on daytime performance, we decided to use an inert placebo, non-fermented milk, to evaluate the contribution of both LcS and its metabolites to any health benefits for the endpoint. The nutritional content, color, flavor, taste, and pH of the non-fermented placebo milk were adjusted to match the test beverage. The placebo was made from the same ingredients as the test beverage, except that lactic acid was added to match the acidity [31], and the identical plastic bottle was used for storage and the provision of the test beverage and the placebo. The beverages were hand-delivered to each participant weekly and stored at 0–10 °C.

### 2.2. Study Design and Participants

**Figure 1:**
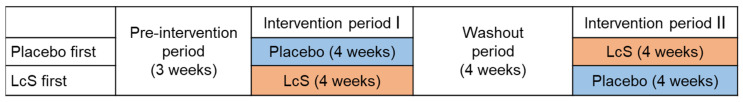
Study design. LcS, *Lacticaseibacillus paracasei* strain Shirota.

This study was conducted in Kunitachi-shi, Tokyo, from August to December 2021 as a double-blind, randomized, crossover, placebo-controlled study. The study protocol was registered at the UMIN (University Hospital Medical Information Network) Clinical Trials Registry (ID: UMIN000044852). The study comprised a 3-week pre-intervention period, a first 4-week intervention period, a 4-week washout period, and a second 4-week intervention period (Figure 1). Healthy male and female office workers were recruited for this study. Because little information was available on the impact of probiotics on daytime performance, the present study is seen as an exploratory study in this field, and no formal sample size calculation was performed. We set the sample size to 12 in total by considering the handling capability of the EEG system, which was to be used only on weekdays during a specified period. There are several reports that cognitive abilities decline with age [34,35,36] and lack of sleep [37,38]. It has also been reported that the reaction times of the go/no-go task decline with age older compared to 40 years [35]. Based on these observations, office workers between 40 and 59 years old who were aware of impaired sleep quality were chosen as the participants. Those meeting any of the following criteria were excluded from the study: a history of sleep-related illnesses, use of medicines or health foods that could influence sleep, smoking habits, allergies to milk or soy, placement of an artificial cardiac pacemaker, skin disorders on the face or head, severe dry eye symptoms, and participation in other clinical trials during the study period. Finally, 5 males and 7 females were enrolled and were divided into the following two groups: one group was treated with the placebo and LcS in that order, and the other group was treated in the reverse order. Simple randomization was performed by assigning random numbers from a random number table to the groups. The codes of the treatments were assigned by personnel not involved in the study, and participants and those giving treatment or assessing outcomes were blinded to the treatment allocation.

### 2.3. Questionnaires

The daytime mood, including perceived sleepiness, physical fatigue, motivation, attention, and optimism, was surveyed using a visual analog scale (VAS) ranging from 0 mm for “severely unfavorable” daytime performance to 100 mm for “supremely favorable”. Sleepiness was also evaluated on a 9-point Likert scale using the Karolinska Sleepiness Scale (KSS) [39]. Participants completed the questionnaires at an arbitrary time in the morning (8:00–11:00) and in the afternoon (15:00–18:00) every day for 5 weekdays during the last week of each period. Information on sleep habits and stress levels was also collected via retrospective self-assessments for the previous 1-month period before intervention using the Pittsburgh Sleep Quality Index (PSQI) [40] and the Japanese version of the Perceived Stress Scale (JPSS) [41], respectively.

### 2.4. Daytime EEG and HRV Measurement

EEG and HRV measurements were conducted in a shielded room during the last week of each period: in the pre-intervention period and in intervention periods I and II. Participants underwent measurements on any day from Tuesday to Thursday in the morning (8:00–11:00) and in the afternoon (15:00–18:00). Each measurement required about 1 h, including preparation and cleanup, and the day and time of each individual’s measurements were matched between the experimental periods. We instructed the participants not to consume alcohol for at least 24 h before the measurement and caffeinated beverages on the day of measurement and confirmed the participants’ adherence to the instructions before the measurement.

EEG signals were recorded with Ag/AgCl electrodes placed on the scalp at the Fz, Cz, and Pz positions according to the International 10–20 System. Two electrodes placed on both ears were regarded as a reference for the scalp electrodes, and two forehead electrodes served as the ground and system reference. To aid in the elimination of data obtained during eye movements or blinks, an electrooculogram (EOG) was recorded with an electrode placed 2 cm lateral to the lateral canthus of the left eye and 2 cm above the upper edge of the left orbit. Electrodes were also attached to the left arm to monitor the electrocardiogram (ECG) for HRV analysis. EEG and ECG were recorded using a Polymate^®^ biological signal recording device (Miyuki Giken Co., Ltd., Tokyo, Japan) with a sampling rate of 1000 Hz; the impedances of all electrodes were kept below 10 kΩ.

**Figure 2:**
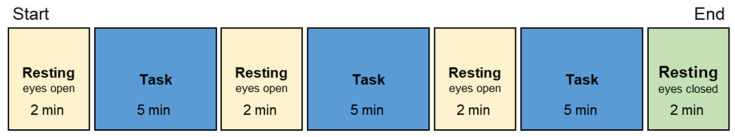
Experimental procedure for electroencephalogram and heart rate variability measurement.

The procedure for each measurement is shown in Figure 2. The participants performed the whole task seated on an armchair, fixating their eyes on a point directly in front of them at a 0.5 m distance. During a 5 min task period, participants performed an auditory oddball task with a 2-tone paradigm. The procedures were compliant with the guidelines of the Japanese Society of Clinical Neurophysiology [42], and a target tone (2000 Hz) and a standard tone (1000 Hz) were randomly emitted every 2.0 s with a 100 ms duration for each, 150 times in total, with appearance rates of 20% and 80%, respectively, using the Stimuli Output Sequencer Program v. 2009-Jan. (NoruPro Light Systems, Inc., Tokyo, Japan). The participants responded by pressing a button only for the target stimulus, and their reaction time was calculated. Finally, EEG was recorded with the eyes closed for 2 min to estimate the basal resting state.

### 2.5. Analysis of EEG and HRV

The MATLAB R2018b mathematical analysis software (MathWorks, Inc., Natick, MA, USA) was used to analyze spontaneous brain waves [43]. Fourier transformation was performed for data removed using a 0.5–30-Hz band-pass filter, and power spectrum analysis was performed in each frequency band (theta wave, 4–8 Hz; alpha wave, 8–13 Hz; beta wave, 13–30 Hz). The event-related potential (ERP) P300 was obtained and analyzed via the arithmetic mean method using the EP Travel Light software v. 2009-Feb. (NoruPro Light Systems, Inc.). When P300a and P300b were observed, P300b was selected, and when three or more peaks were observed, it was considered inconclusive. An analysis program based on the MemCal method using the RR Interval Analysis software v. 2009-Feb. (NoruPro Light Systems, Inc.) was used to analyze heart rate and HRV. LF components at 0.04–0.15 Hz, reflecting sympathetic and parasympathetic nerve activity, and HF components at 0.15–0.4 Hz, reflecting parasympathetic nerve activity, were analyzed, and the ratio of the LF/HF parameters was calculated as an index of sympathetic nerve activity [12].

### 2.6. Statistical Analysis

The order effect and time effect of the crossover test were calculated from repeated-measures analysis of variance in the pre-intervention period, intervention period I, and intervention period II. Variables were subjected to the assessment of treatment efficacy if they did not have any significant order and time effects (*p* ≥ 0.05). The pairwise comparison between the placebo and LcS intake periods was performed using the Wilcoxon rank sum test. Fisher’s exact test was used to compare the proportions in participant attributes. To assess statistical associations between variables, Spearman’s rank correlation coefficient (*ρ*) was calculated for differences in the data between the placebo and LcS periods. Spearman’s rank correlation coefficient (*ρ*) was also calculated for the data at the pre-intervention period. Due to the exploratory character of the study and the clear definition of a single primary outcome parameter, no correction for multiplicity testing was applied. The significance level was set at 0.05, with less than 0.05 considered significant. The EZR and R v. 4.2.0 statistical analysis software (R Foundation for Statistical Computing, Vienna, Austria) were used for the analysis.

## 3. Results

### 3.1. Characteristics of Participants

The participants were 12 healthy male and female office workers who consented to participate and were divided into the following two groups: one group received the placebo treatment first, while the other group received the LcS treatment first. There were no significant differences between the two groups in terms of their age, height, weight, JPSS, PSQI, and number of complaints about sleep (Table 1). The rate of compliance for test beverage consumption was calculated as the percentage of the actual intake over a defined period, and the results were 99.7% in the placebo treatment and 100% in the LcS one. No adverse events related to the test beverage intake were identified. Blinding was confirmed at the end of the study based on responses when the participants were asked to guess which beverage they received first.

### 3.2. Perceived Mood

**Figure 3:**
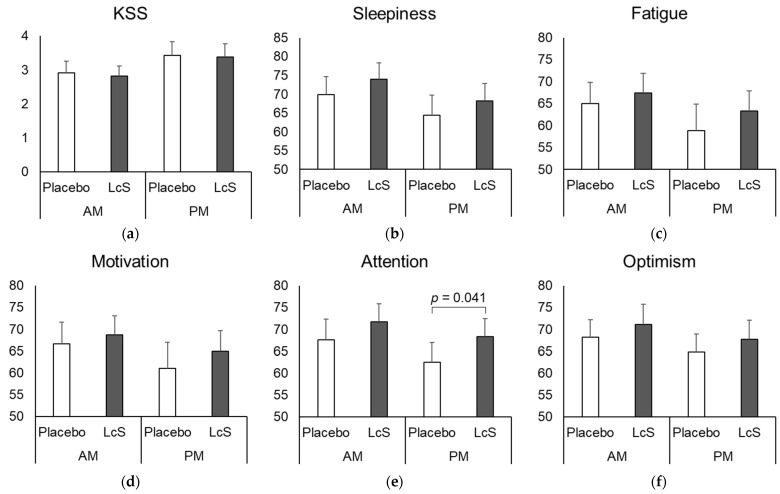
Impact of *Lacticaseibacillus paracasei* strain Shirota (LcS) intake on daytime mood. Participants completed the questionnaires in the morning (AM) and in the afternoon (PM) every day for 5 weekdays during the last week of each 4-week placebo and LcS intervention period. Sleepiness was evaluated with the Karolinska Sleepiness Scale (KSS) (**a**). Perceived sleepiness (**b**), physical fatigue (**c**), motivation (**d**), attention (**e**), and optimism (**f**) were also surveyed using a visual analog scale (VAS) from 0 mm for “severely unfavorable” daytime performance to 100 mm for “supremely favorable”. Values are expressed as the means + standard error (*n* = 12). The Wilcoxon rank sum test was used for the comparison between treatments.

Daytime mood was assessed using the KSS and VAS items asking about perceived sleepiness, fatigue, motivation, attention, and optimism. The scores were generally better during the LcS intake period than during the placebo intake period in both the morning and afternoon (Figure 3). The VAS score of attention in the afternoon was significantly higher during the LcS intake period than during the placebo intake period (Figure 3e, *p* = 0.041).

### 3.3. EEG

**Figure 4:**
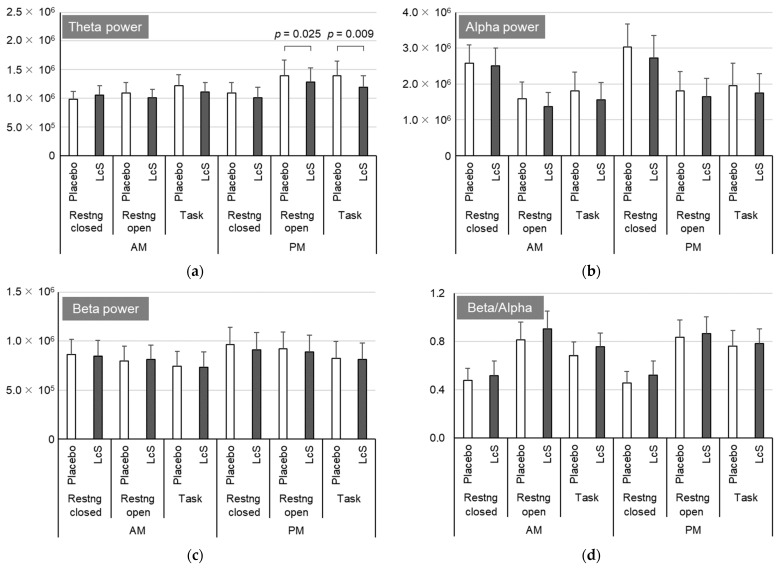
Impact of *Lacticaseibacillus paracasei* strain Shirota (LcS) intake on electroencephalography (EEG). EEG was measured during the last week of each 4-week placebo and LcS intervention period, and the power spectrum (μV^2^/min) of theta waves (4–8 Hz) (**a**), alpha waves (8–13 Hz) (**b**), beta waves (13–30 Hz) (**c**), and the beta/alpha ratio (**d**) were calculated during the resting state (eyes open and closed) and during the task both in the morning (AM) and the afternoon (PM). Values are expressed as the means + standard error (*n* = 12). The Wilcoxon rank sum test was used for the comparison between treatments.

There was no significant difference in the alpha, beta, or beta/alpha ratio between the treatments, both during the resting state and during the tasks (Figure 4). Theta power (μV^2^/min) observed during the resting state with the eyes open and during the oddball task in the afternoon was significantly lower during the LcS intake period than during the placebo intake period (*p* = 0.025 and 0.009, respectively).

Table S1 shows the button-press reaction time and the ERP P300 latency to the target stimulus in auditory oddball tasks. The reaction time during the LcS intake period tended to be shorter when compared to the placebo intake period in both the morning and the afternoon, although this difference was not statistically significant. The efficacy assessment of the ERP P300 latency was available only in the morning measurements because, for the afternoon measurements, we were able to determine the P300 in only 8 participants for both intervention periods (10 in the LcS intake period and 8 in the placebo). The morning P300 latency was shorter during the LcS intake period than during the placebo intake period.

### 3.4. HRV

Table 2 shows the heart rate and autonomic nerve indices calculated from HRV. Heart rate was lower during the LcS intake period than during the placebo intake period, regardless of the time (AM/PM) and activity (resting/task). No significant difference was observed in either the LF/HF ratio (an index of sympathetic nerve activity) or the HF component (parasympathetic nerve activity), except for the LF/HF ratio during the afternoon oddball task.

### 3.5. Relationship between the Perceived Mood and Physiological Parameters

**Figure 5:**
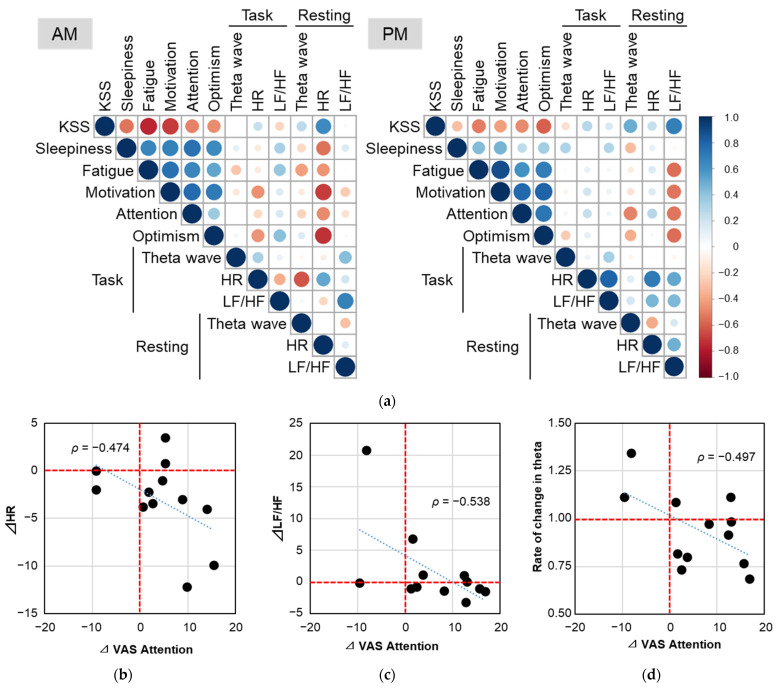
Correlation analysis between perceived daytime mood and physiological indices. The correlation matrix was based on Spearman’s rank correlation coefficient (*ρ*) between the perceived mood and physiological indices using the difference in values of 12 participants between the placebo and *Lacticaseibacillus paracasei* strain Shirota (LcS) intervention periods (**a**). Color intensity and the size of circles are proportional to correlation coefficients. Relationships between the visual analog scale (VAS) attention score and morning heart rate (HR), (**b**) afternoon ratio of low-frequency (LF) components to high-frequency (HF) components (LF/HF), (**c**) and afternoon electroencephalogram theta power (**d**). Plots from 12 participants and the fitted linear trendline (dotted blue line) are shown, and data lying on dashed red lines indicate no difference between the periods. AM, morning; KSS, Karolinska Sleepiness Scale; PM, afternoon.

We analyzed the relationship between the perceived mood and physiological indices using the differences between the placebo and LcS interventions (LcS-placebo, Figure 5a). The LcS/placebo ratio was used in EEG data. In the morning, heart rate during the resting state with the eyes closed was associated with the KSS (*ρ* = 0.636; 95% CI 0.193 to 0.880), sleepiness (−0.545; −0.905 to 0.139), motivation (−0.699; −0.914 to −0.156), fatigue (−0.441; −0.871 to 0.169), attention (−0.474; −0.862 to 0.319), and optimism (−0.730; −0.939 to −0.291). In the afternoon, LF/HF during the resting state with eyes closed was associated with the KSS (0.687; 0.256 to 0.889), motivation (−0.531; −0.978 to 0.162), fatigue (−0.552; −0.927 to 0.028), attention (−0.538; −0.865 to 0.036), and optimism (−0.552; −0.900 to 0.068). As for EEG, the theta wave during the resting state with the eyes closed was associated with the KSS (0.493; −0.228 to 0.919) and attention (−0.497; −0.913 to 0.235). The relationships between the theta wave, heart rate, LF/HF, and attention are also shown graphically (Figure 5b–d). These relationships indicate that physiological indices reflect subjective indices.

## 4. Discussion

We investigated whether LcS affects the daytime performance of healthy office workers with sleep complaints and observed that LcS intake suppresses the decline in perceived attention in the afternoon. This result is considered to be supported by the fact that other VAS items (sleepiness, fatigue, motivation, and optimism) tended to be better during the LcS intake period. All items of VAS were lower in the afternoon than in the morning, suggesting an overall decline in performance in the afternoon. The VAS score of attention in the afternoon was significantly higher during the LcS intake period than during the placebo intake period. The VAS attention score in the afternoon during the LcS intake period was similar to that of the placebo intake period in the morning, indicating that the effect size of LcS was comparable to the range of diurnal variation.

During the LcS intake period, the EEG theta power that appeared during the resting state (eyes open) and when performing tasks in the afternoon was significantly lower than that during the placebo intake period. Correlation analysis showed that the changes in daytime attention with LcS intervention were associated with the changes in theta power in the resting state (eyes closed) in the afternoon (*ρ* = −0.497). These results indicate that LcS increases the arousal level both subjectively and objectively. Spontaneous brain waves are constantly observed, and the degree of arousal is estimated by the intensity of beta, alpha, theta, and delta waves (classified by frequency). Alpha and beta waves are observed in the wakeful state [9], and theta waves increase when the degree of wakefulness is reduced. The beta/alpha ratio during wakefulness has also been widely used as an index of attention and stress in previous reports [44]. The beta/alpha value tended to be higher during LcS intake than with the placebo, supporting an increased arousal level, although this difference was not statistically significant.

The shortened latency of the ERP P300 also supports the results, indicating improved perceived attention. ERP P300 latency is believed to indicate the attentional state of the brain. Prolonged P300 latency is related to aging [34], depression [45], and dementia [46]. The ingestion of polyunsaturated fatty acids [47] or caffeine [48] can shorten P300 latency. However, the ERP P300 peak measured in the afternoon was not detectable in this study, suggesting that P300 characterizes brain traits under good conditions but may not be suitable for assessing brain status under poor conditions. For example, a decrease in P300’s amplitude has been reported during sleep deprivation [49]. However, the fact that the LcS intervention showed a tendency for a shorter button-press reaction time in our task provides data supporting increased attention.

Heart rate during the LcS intake period was lower than that during the placebo intake period, regardless of the time of day or if the individual was resting/performing a task, with a significant difference in the resting heart rate (with eyes closed) in the morning. Moreover, changes in heart rate with closed eyes in the morning were correlated with improved mood (positively for the KSS, negatively for motivation and optimism). A reduction in heart rate induced by LcS intervention has also been observed after the intake of a hot water extract of LcS [50], which is consistent with our results. The LF/HF values during the afternoon oddball task were significantly lower during the LcS intake period. The reduction in heart rate and LF/HF with LcS in the present study supports the finding that LcS affects the autonomic nervous system because HRV has been considered a product of emotional response or stress exerted via the autonomic nervous system. These neurological responses are consistent with the ability of LcS to attenuate a stress-induced rise in cortisol in the academic test stress model [30,31,32]. In an animal study, the intragastric administration of LcS stimulated gastric vagal afferent activity in a dose-dependent manner [32], and it has been shown to suppress stress-induced sympathetic nerve activity via signal transmission through the gastric vagal afferent [51,52]. The results of these animal and human experiments suggest that LcS acts via the autonomic nervous system to improve performance. LcS may also affect mood because several probiotics have been reported to normalize anxiety-like behavior via the vagus nerve [53,54].

Only individuals with sleep complaints were included in this study, and stress was not taken into consideration. The stress levels of the participants enrolled in this study were not very high in terms of JPSS values. LcS has been shown to prevent the worsening of stress and sleep quality under academic stress [30,31,32,33], but the effect on stress and sleep may be mild in people with low-stress levels, as in this study. When we analyzed the relationship between perceived mood and other parameters in the pre-intervention period, a high negative correlation was confirmed between the stress state and perceived mood in the morning and afternoon, and the degree of correlation was stronger than that with sleep (Table S2). Our results are consistent with reports that mental and physical stress and sleep disturbances directly and indirectly affect job performance [4,55]. The Japanese Ministry of Health, Labor, and Welfare has reported that half of the Japanese population lives with worry or stress and that one-fourth of working-age people have sleep complaints [56]. The effects of LcS on work performance may be captured more clearly by targeting participants with high-stress levels.

The present study must be seen as an exploratory study in this field, and thus, no formal sample size calculation was performed. We set the sample size to 12, considering the handling capability of the EEG system, but this may be insufficient for making strong conclusions. To eliminate this limitation, we adopted a crossover study design. The crossover design has high power and statistical efficiency, and it is possible to obtain an estimate with the same level of accuracy as a parallel design, even with a smaller number of participants [57]. We confirmed that the crossover trial was conducted properly without any time or order effect. Another concern was the selection of a placebo and its impact when used in the crossover study. We selected non-fermented milk as a placebo, the nutritional content, color, flavor, taste, and pH of which were adjusted to match the test beverage, but it was practically impossible to make the characteristics of the non-fermented milk have complete correspondence with the fermented one, considering the wide variety of metabolites produced by microbial fermentation. Blinding was assured by confirming that the participants could not guess at the end of the study which treatment they received first. However, it cannot be concluded that subtle differences in flavor and texture between the test beverage and the placebo did not have any impact on the study outcomes. We believe that the current study basically provides reliable results on the effect of LcS on daytime performance, and further studies using a randomized, placebo-controlled, double-blind, parallel-group study design with a larger sample size are needed to address such limitations.

## 5. Conclusions

Our results suggest that LcS intervention improves mood when measured as daytime performance indices compared with the placebo. Furthermore, the daytime perceived mood indices were associated with physiological parameters such as EEG and autonomic nerve activity data.

## Figures and Tables

**Table 1:** Characteristics of the participants.

	Placebo First	LcS First	Total
Number	2 males, 4 females	3 males, 3 females	5 males, 7 females
Age (years)	47.2 ± 7.8	45.5 ± 5.9	46.3 ± 6.6
Height (cm)	161.75 ± 7.04	166.50 ± 9.89	164.13 ± 8.55
Body weight (kg)	54.66 ± 12.08	58.16 ± 13.22	56.42 ± 12.21
Body mass index	20.71 ± 3.16	20.80 ± 2.89	20.76 ± 2.88
JPSS	18.2 ± 7.5	25.5 ± 11.2	21.8 ± 9.8
PSQI	6.7 ± 1.6	7.3 ± 1.8	7.0 ± 1.7
Complaints about sleep			
Sleep latency	1/6	0/6	1/12
Wake after sleep onset	5/6	4/6	9/12
Waking up too early	3/6	3/6	6/12
Sleep quality	1/6	2/6	3/12
Sleepiness on rising	2/6	2/6	4/12
Daytime sleepiness	3/6	4/6	7/12
Sleep duration	2/6	2/6	4/12

Values are expressed as the means ± standard deviation (quantitative variables) or proportions (complaints about sleep). *Lacticaseibacillus paracasei* strain Shirota (LCS); JPSS, Japanese Perceived Stress Score; PSQI, Pittsburgh Sleep Quality Index.

**Table 2:** Impact of LcS intake on the following HRV metrics: heart rate and autonomic nerve indices.

	Time	Treatment	HR (bpm)		LF/HF		HF (ms^2^)
Resting (Eyes closed)	AM	Placebo	67.8 ± 3.1		4.13 ± 1.20		321.1 ± 105.9
		LcS	64.7 ± 3.0	*	3.60 ± 1.12		200.4 ± 37.1
	PM	Placebo	68.0 ± 3.0		3.52 ± 1.45		390.2 ± 215.1
		LcS	66.9 ± 4.0		5.28 ± 3.16		279.3 ± 103.7
Resting (Eyes open)	AM	Placebo	67.5 ± 2.5		6.69 ± 1.86		307.9 ± 129.4
		LcS	66.5 ± 2.8		4.73 ± 1.07		295.5 ± 90.8
	PM	Placebo	68.5 ± 2.7		5.67 ± 1.50		348.0 ± 142.2
		LcS	67.0 ± 3.4		6.59 ± 2.50		352.5 ± 96.4
Task	AM	Placebo	67.4 ± 2.6		4.55 ± 1.33		296.5 ± 63.7
		LcS	65.9 ± 2.9		3.32 ± 1.00		430.2 ± 198.4
	PM	Placebo	68.0 ± 3.1		4.64 ± 1.32		420.9 ± 203.2
		LcS	66.4 ± 3.4		3.24 ± 0.87	*	289.8 ± 70.8

Heart rate variability (HRV) was measured during the last week of each 4-week placebo and *Lacticaseibacillus paracasei* strain Shirota (LcS) intervention period, and heart rate (HR), the ratio of low-frequency (LF) components to high-frequency (HF) components, and HF were calculated in the resting state (with the eyes closed and open) and during the auditory oddball task. Values are expressed as the means ± standard error (*n* = 12). The Wilcoxon rank sum test was used for the comparison between treatments (*, *p* < 0.050). AM, morning; PM, afternoon.

## Data Availability

The data are not available due to the nature of this research. Participants of this study did not agree for their data to be shared publicly.

## Supplementary Materials

The following supporting information can be downloaded at: https://www.mdpi.com/article/10.3390/nu15245119/s1, Table S1: Effect of LcS on reaction time and ERP P300 latency in the auditory oddball task. Table S2: Correlation of the daytime mood with other indicators during the pre-intervention period.
