# Supplementary material for: Effects of Lacticaseibacillus paracasei Strain Shirota on Daytime Performance in Healthy Office Workers: A Double-Blind, Randomized, Crossover, Placebo-Controlled Trial
Source: Nutrients. 2023 Dec 15;15(24):5119. doi: 10.3390/nu15245119 (PMC10745872; doi:10.3390/nu15245119)
Supplement: Supplementary file 1 [file nutrients-15-05119-s001.zip › nutrients-2720003-supplementary.pdf]

Table S1. Effect of LcS on reaction time and ERP P300 latency in the auditory oddball task

Item		Time	Placebo	LcS	
Reaction time (ms)		AM	365.9 ± 24.2	349.2 ± 27.0	
		PM	381.6 ± 31.3	356.0 ± 31.9	
ERP P300 latency (ms)	Fz	AM	330.7 ± 8.0	323.4 ± 9.7	*
	Cz	AM	329.2 ± 9.3	321.3 ± 9.2	*
	Pz	AM	339.3 ± 8.5	324.9 ± 9.1	*

Reaction time and event-related potential (ERP) latency for the target stimulus in the auditory oddball task were measured during the last week of each 4-week placebo and Lacticaseibacillus paracasei strain Shirota (LcS) intervention period. Values are expressed as means ± standard error (Reaction time: n = 12, ERP P300 latency: n = 10). The Wilcoxon rank sum test was used for the comparison between treatments (*, $p < 0.050$). AM, morning; PM, afternoon.

Table S2. Correlation of the daytime perceived mood with the other indices during the pre-intervention period.

Time	Item			KSS	VAS				
					Sleepiness	Fatigue	Motivation	Attention	Optimism
AM	EEG	Task	Theta	−0.149	0.133	−0.007	−0.004	0.007	0.070
			Beta/alpha	−0.109	0.566	0.608 *	0.559	0.622 *	0.413
		Resting eyes-open	Theta	−0.260	0.144	0.049	0.025	0.039	0.210
			Beta/alpha	−0.056	0.587 *	0.636 *	0.566 *	0.622 *	0.469 *
		Resting eyes-closed	Theta	−0.179	−0.147	−0.245	−0.231	−0.196	−0.119
			Beta/alpha	−0.418	0.503	0.531	0.545	0.629 *	0.503
	HRV	Task	HR	−0.091	−0.028	−0.102	−0.137	−0.165	−0.151
			HF	−0.411	0.105	0.007	0.126	0.063	0.063
			LF/HF	0.077	0.175	0.252	0.098	0.189	0.259
		Resting eyes-open	HR	−0.095	−0.014	−0.084	−0.133	−0.154	−0.105
			HF	−0.186	−0.252	−0.294	−0.196	−0.224	−0.203
			LF/HF	0.354	−0.308	−0.196	−0.371	−0.308	−0.231
		Resting eyes-closed	HR	−0.168	0.042	0.007	−0.084	−0.028	−0.056
			HF	0.056	−0.399	−0.420	−0.406	−0.441	−0.336
			LF/HF	0.242	0.154	0.238	0.175	0.224	0.063
	JPSS			0.497	−0.760 **	−0.729 **	−0.718 **	−0.767 **	−0.676 **
	PSQI			0.199	0.054	0.140	0.064	−0.054	0.097
PM	EEG	Task	Theta	−0.074	0.021	0.077	0.280	0.123	0.070
			Beta/alpha	−0.346	0.622 *	0.685 *	0.476	0.490	0.531
		Resting eyes-open	Theta	−0.212	0.042	0.133	0.343	0.270	0.140
			Beta/alpha	−0.370	0.629 *	0.622 *	0.413	0.462	0.462
		Resting eyes-closed	Theta	−0.138	−0.098	0.007	0.245	0.144	0.035
			Beta/alpha	−0.374	0.503	0.671 *	0.608 *	0.687 *	0.615 *
	HRV	Task	HR	−0.233	−0.098	−0.077	−0.140	0.070	−0.140
			HF	0.360	−0.811 **	−0.706 *	−0.566	−0.483	−0.615 *
			LF/HF	−0.268	0.273	0.210	0.161	0.214	0.147
		Resting eyes-open	HR	−0.268	−0.021	0.035	−0.028	0.123	−0.049
			HF	0.247	−0.657 *	−0.601 *	−0.503	−0.410	−0.545
			LF/HF	−0.046	0.126	0.084	0.042	0.018	0.063
		Resting eyes-closed	HR	−0.346	0.021	0.007	−0.056	0.137	−0.098
			HF	0.367	−0.783**	−0.678 *	−0.559	−0.504	−0.587 *
			LF/HF	−0.200	−0.123	−0.070	−0.161	−0.037	−0.144
	JPSS			0.472	−0.837 ***	−0.753 **	−0.753 **	−0.732 **	−0.771 *
	PSQI			0.211	−0.032	−0.129	−0.279	−0.280	−0.043

Spearman's rank correlation coefficient (ϱ) was calculated to assess statistical associations between the daytime perceived mood and the other indices measured during the pre-intervention period (n = 12, * $p < 0.050$, ** $p < 0.010$, *** $p < 0.001$). AM, morning; EEG, electroencephalogram; HF, high-frequency components; HR, heart rate; HRV, heart rate variability; JPSS, Japanese Perceived Stress Score; KSS, Karolinska Sleepiness Scale; LF/HF, index of sympathetic nerve activity; PM, afternoon; PSQI, Pittsburgh Sleep Quality Index; Task, auditory oddball task; VAS, visual analog scale.
